# Key stakeholders’ perspectives of illicit drug use and associated harms in the Northern Territory of Australia

**DOI:** 10.1186/s12954-024-01092-w

**Published:** 2024-09-20

**Authors:** Bryce Brickley, Samuel Moore, Noemi Tari-Keresztes, Anthea Brand, Madeleine Bower, Jason G. Bonson, Alice McEntee, Ashlea J. Bartram, Nataly Bovopoulos, Skye McPhie, Craig Martin, Cassandra Wright, Jacqueline Bowden, James A. Smith

**Affiliations:** 1 College of Medicine and Public Health, Flinders University, Flinders Health and Medical Research Institute, Darwin, NT Australia; 2 College of Medicine and Public Health, Flinders University, Flinders Health and Medical Research Institute, Alice Springs, NT Australia; 3 College of Medicine and Public Health, Flinders University, Flinders Health and Medical Research Institute, Katherine, NT Australia; 4 College of Medicine and Public Health, Flinders University, Flinders Health and Medical Research Institute, Adelaide, SA Australia; 5 National Centre for Education and Training on Addiction, Flinders University, Bedford Park, SA Australia; 6 Alcohol and Drug Foundation, Melbourne, VIC Australia; 7 Menzies School of Health Research, Charles Darwin University, Darwin, NT Australia; 8 Centre for Alcohol Policy Research, La Trobe University, Melbourne, VIC Australia; 9 Burnet Institute, Melbourne, VIC Australia

**Keywords:** Illicit drugs, Harm reduction, Quality improvement, Needs assessment, Evidence gaps, public health

## Abstract

**Background:**

In the Northern Territory (NT) of Australia, there are significant evidence gaps about illicit drug use and harms, despite having established monitoring and reporting systems. This paper reports on illicit drug use, associated harms, contributing factors, service needs and priorities in the NT from the perspective and experiences of key stakeholders engaged in providing services for, or advocating on behalf of, people who use illicit drugs in the NT.

**Methods:**

Face-to-face and online qualitative interviews were conducted with stakeholders across urban and remote locations in the NT. Key stakeholders were service providers, including acute and primary care clinicians, representatives of Aboriginal community controlled health organisations, lived experience advocates, peak body representatives and public health executives. Qualitative data were analysed thematically.

**Results:**

Four researchers interviewed 21 participants across urban (62%), and remote areas (38%) of the NT. Themes identified were: (1) Illicit drug use and harms are diverse and distinct; (2) Client support needs are complex and influenced by co-morbidities, socio-demographic and cultural factors; (3) Priority population sub-groups need targeted strategies; (4) Local service strengths can be further developed and enhanced; (5) Local services need better resourcing; (6) Invest in progressive legislative and policy reforms; and (7) Improve routine monitoring and evaluation.

**Conclusions:**

Key stakeholders described illicit drug use, harms and contributing factors, which provided insights into the local challenges. Participants emphasised that clients have complex care needs, and further investment into targeted strategies are required to improve service engagement with priority groups. Service needs included greater understanding the role of dual diagnosis and its implementation and enhancing integrated and collaborative care in both primary health and acute care contexts. The voices of people with lived experience captured in this paper must inform local strategy and policy development relating to illicit drug use, in alignment with national strategy.

**Supplementary Information:**

The online version contains supplementary material available at 10.1186/s12954-024-01092-w.

## Introduction

Australia has established monitoring and reporting systems for illicit drug use, including the National Drug Strategy Household Survey Data (NDSHS) [1], Illicit Drug Reporting System (IDRS) [2], Ecstasy and Related Drugs Reporting System (EDRS) [3], and National Wastewater Drug Monitoring Program [4]. Australia’s largest population survey on drug use, the NDSHS report that 18% of Australians aged ≥ 14 had illicitly used a drug in the past 12 months [1]. Regional disparities exist, with the Northern Territory (NT) having the highest prevalence of illicit drug use in the past 12 months among all Australian states and territories at 25% [1]. According to the NDSHS, the most commonly used illicit drugs in the NT are cannabis (18.9%), cocaine (4.2%) and ecstasy (2.2%) [1, 2]. The NT also has the highest levels of alcohol consumption compared to other Australian states and territories [4], and high rates of alcohol-related injury and harms [5, 6]. Furthermore, the World Drug Report 2023 outlined that 6% of people aged 15–64 had used a drug in the past 12 months, up 23% from a decade earlier, highlighting the growing challenges associated with substance use worldwide [7]. The increasing prevalence of illicit drug use highlights the need for evidence-based strategies to address its broad social, health and economic consequences.

The NT is the least populous jurisdiction in Australia, home to less than 1% of the country’s population (approximately 250,000 people) [8]. The NT has a higher level of disadvantage compared to other Australian regions, with 13 local government areas falling within the top 10 percentile for relative disadvantage [9]. Quantitative data indicates that NT residents engaging in illicit drug use exhibit disproportionately high rates of mental health conditions, criminal involvement and drug-related hospitalisations [10]. Substance use disorders, suicide and mental health conditions collectively contribute to approximately 36% of the total burden of disease in the NT, three times the national average [11]. Furthermore, approximately 31% of NT residents identify as Aboriginal and/or Torres Strait Islander [12], and this cohort are significantly over-represented in alcohol and other drugs (AOD) treatment services [13]. To support the development of locally and culturally relevant evidence-based strategies for preventing harms, contextually relevant research is needed to deepen our understanding of illicit substance use, its associated harms, and protective factors.

Within the NT AOD sector, existing research is primarily focused on alcohol use, harms, policies and treatment [14–18], leaving a research gap on illicit drug use and associated harms. Moreover, limitations exist in monitoring and reporting systems for illicit drug use and harms in the NT, such as limited participant numbers and representativeness, inadequate engagement with priority populations, and poor geographical coverage. Challenges with research and evaluation sampling in the NT are exacerbated by the high cost of rural and remote engagement [19] and the time needed to build trust for AOD research participation. For instance, the NT specific EDRS drug trends findings were not published in 2022 and 2023 due to insufficient sample size [3]. Geographical coverage challenges are evident, with only one regional site in the National Wastewater Drug Monitoring Program covering less than 30,000 people [4], despite over 40% of the NT population living in non-urban areas [8]. Inadequate data collection in the NT can hinder evidence-based care and treatment, delay illicit drugs policy development and implementation, and inhibit strategic resourcing.

Qualitative methods can engage lived experience voices, offering valuable insights to explore deeper meanings and understandings within a given context and/or setting, thereby building on existing quantitative findings. Globally, qualitative approaches have explored illicit drug use experiences and service needs, informing service planning and strategies in specific contexts [20, 21]. Understanding illicit drug use patterns and harms contextually can inform program and service development to address the needs of at-risk populations [22, 23]. This study aims to explore perspectives and experiences of key stakeholders in the NT regarding illicit drug use, associated harms, contributing factors, and service needs and priorities.

## Methods

### Methodological overview

A qualitative descriptive approach [24], a form of naturalistic, contextually relevant inquiry, was employed within the constructivist paradigm [25]. Reporting followed the Consolidated criteria for reporting qualitative research (COREQ) checklist (Supplementary File 1) [26]. Ethics approval was obtained from the Human Research Ethics Committee of NT Department of Health and Menzies School of Health Research (Ref: 2023–4545). The research question was: “What are the perspectives of stakeholders in the NT in relation to illicit drug use, associated harms, contributing factors, service needs and priorities?”

### Selection and recruitment of study population

Eligible participants were adults (≥ 18 years) with lived experience of illicit drug use and/or direct experience working with or advocating on behalf of people who use illicit drugs in the NT. The NT Lived Experience Network (NTLEN), consisting of people with lived experience of mental health, AOD and related challenges, was engaged to incorporate consumer voices. We employed a purposive sampling approach [27] to identify people with unique and deep insights in relation to the research question. To do this, we leveraged the research team’s trusted relationships with local AOD stakeholders their deep understanding of NT AOD services and policy. Researchers (BB, JS, SM, AB, MB, JGB, NTK) identified relevant organisations and individuals in consultation with an expert advisor (CW). Potential participants were members of the NTLEN, representatives of NT AOD peak harm minimisation bodies and their member organisations, Aboriginal community controlled organisations, other AOD service providers, including a range of non-government and government employed clinicians and/or AOD workers, health professionals, policy staff, and NT public health system executives. Invitations to participate were sent via email and one-on-one or group interviews were scheduled with each interested stakeholder.

### Interview protocol

The interview commenced with an Acknowledgement of Country, followed by a short briefing and interview questions in accordance with the interview guide (Table 1). An interview guide was developed (researchers: BB, JS, JGB, AJB, NTK, JB, CW) using a logic model, where underpinning ideas formed key questions, supported by additional probing questions. The interview guide was then tested in a one-on-one pilot interview with a representative from the Association of Alcohol and Other Drugs Agencies NT. After this pilot interview, the interview guide was modified to include the concepts of illicit drug workforce needs, program/service monitoring and evaluation, and probing questions about trauma-informed care and the socio-cultural determinants associated with illicit drug use and harms.

**Table 1:** Semi-structured interview guide

Question Logic	Main Interview Questions	Potential Probing Topics and Prompts	Duration
Gain a better understanding of the harms associated with illicit drug use in the NT	1. What is your understanding of the current state of illicit drug use across the NT? 2. What do you perceive to be the harms associated with illicit drug use in the NT? 3. In which groups/areas do you see these harms? 4. What are the reasons why these harms might be seen more in these particular areas and/or populations?	• Probe to ensure specific substance use is discussed: cannabis (most common in the NT), cocaine, ecstasy/MDMA, meth/amphetamine, non-prescribed use of pharmaceuticals e.g. opioids; other illicit substances; and any new or emerging drugs of concern • Probe around the concept of ‘harms’. It should be discussed in harms to the individual who is using the drug and harms to other aspects e.g., family, social, and community. • Probe for which primary drug of concern is linked to harms discussed. • Probe as to whether there are any different patterns of needs in different towns/regions of the NT. • Probe re: impact of/on social and cultural determinants of health.	~ 15–20 min
Explore needs and priorities to reduce harms associated with illicit drug use	5. What support is currently in place for those impacted by harms of Illicit drug use? 6. What are the strengths and weaknesses of these supports/services? 7. Are the current services servicing the client groups most at need? 8. What are the needs to support the monitoring and evaluation of these services and programs? 9. What would be the best investments or critical priorities for future illicit drug harm reduction? 10. What are the health workforce needs to address illicit drug harms in the NT?	• Probe for services/programs/policies focused on harm reduction, demand reduction or supply reduction. • Probe for priorities across specific use of substances. • Probe for treatment of comorbidities and adequacy of services supporting socio-cultural determinants of illicit drug use. • Probe for services and investments targeting types of use: non-harmful use/recreational use vs. dependent use.	~ 15–20 min
Explore and plan for specific areas of need for future programs supporting Aboriginal and Torres Strait Islander people and other priority populations	11. The NT has a large Aboriginal and Torres Strait Islander population, who are disproportionately impacted by illicit substance use. How might future services best support their needs? 12. What key considerations are needed to ensure future illicit drug harm minimisation programs/services engage Aboriginal and Torres Strait Islander people and communities successfully? 13. Are there other priority populations that require targeted health services for illicit drug use?	• Probe to ensure specific substance use is discussed. • Improving/integrating cultural safety, trauma informed care and follow-up support mechanisms • Probe for other high-risk client populations e.g. people in contact with the criminal justice system; LGBTIQA+; young men; people living remotely	~ 10–15 min
Provide an opportunity for participants to raise anything not yet said	14. Thank you all for your time. Is there is anything you would like to say that we did not get a chance to discuss today?		~ 5 min

### Data collection

Socio-demographic data were collected using a self-completed form: participant age, gender, location, cultural background, highest education qualification, current illicit drug related role, length of experience in the AOD sector and NT. Qualitative data were collected via one-on-one and dyadic semi-structured interviews [28], conducted either online or face-to-face by four researchers (BB, JS, AB, NTK). A sample size of 9–17 interviews was aimed for in the pursuit of data saturation [29]. This dual approach of online or face-to-face interviews helped enable time-limited stakeholders who were geographically dispersed across the NT to participate in the research. An interviewer was selected from the research team in response to participants’ characteristics and researcher understandings of local context; for example, NTK, a Senior Research Fellow of Lived-Experience Research, facilitated interviews with participants from the NTLEN; and AB, an allied health academic in Central Australia led interviews with clinicians in Central Australia. Oral or written consent was obtained prior to the interview taking place. Lived experience participants received a $50 voucher for their time and contribution. No other participants were offered incentives or reimbursements. Interview length aimed to be 30–45 min (one-on-one) or 45–60 min (dyadic). All interviews were recorded using Microsoft Teams or an audio-recorder and were transcribed verbatim. A de-identified interview transcript was returned to each participant for verification before analysis.

### Data analysis

Participants’ geographic locations were categorised using the Modified Monash Model, a classification that accounts for geographical remoteness and population size [30]. Interview data were analysed in Microsoft Excel using a constant comparative method [31] and thematic analysis [32]. Attride-Stirling’s thematic analysis technique involved: (1) familiarising with the data, (2) coding, (3) theme generation, (4) thematic network construction, (5) network description and summary and (6) pattern integration and report production [32]. One researcher (BB) reviewed interview recordings and checked transcripts before returning them to participants for verification. During this process, the researcher became familiar with the data and identified initial codes. BB and SM collaboratively developed codes and basic themes inductively. This iterative process involved comparing and contrasting data across transcripts, and re-examining emerging codes to ensure relevant, meaningful and accurate interpretation of data. SM’s experience working in AOD treatment contexts in urban and remote NT aided this process. Organising themes were developed by aligning codes and basic themes with conceptual correspondence. As the codebook stabilised, indicating elements of thematic and data saturation [33], the researchers moved on to developing global themes. This required a higher level of abstraction to create overarching themes that encapsulated the study’s findings in relation to the research question. All researchers reviewed and finalised the themes and their integration into the manuscript via a collaborative document.

## Results

### Participants

Of the 51 participants invited to participate, 21 agreed and completed an interview in April or May 2023. Two one-on-one interviews were in person, all other interviews were online via Microsoft Teams videoconference. Of those who actively declined, reasons were due to tight project timeframes and competing work demands. Participants were based in the NT’s most populous locations (62% urban, 38% remote): the capital of Darwin and its surrounds (*n* = 13), Alice Springs (*n* = 5), Katherine (*n* = 2) and Tennant Creek (*n* = 1). On average, participants reported being in their current role or a similar role for nearly 10 years (mean: 9.1 ± 8.9yrs) and living and working in the NT for over 10 years (mean: 13.1 ± 7.9yrs). Participants’ characteristics are shown in Table 2.

**Table 2:** Interview participants

Interview type	Number of Participants	Age (yrs)	Gender	Role	Location	Total Interview duration (mins)
Dyadic and one-on-one	21	Mean: 47 Range: 27–62 NR (*n*= 2)	M = 9 F = 12	Clinician= 6 Service Provider= 3 ACCHO= 1 Other*=7 Lived Experience= 4	Urban NT= 13 *(Darwin and surrounds)* Remote NT= 8 *(Katherine = 2*,* Tennant Creek = 1 Alice Springs = 5)*	658
5 x Dyadic	10	Mean: 49 Range: 35–62 NR (*n*= 1)	M = 5 F = 5	Clinician= 2 Service Provider= 3 Other*=5	Urban NT= 7 (Darwin and surrounds) Remote NT= 3 (Katherine = 2, Alice Springs = 1)	217
11 x One-on-one	11	Mean: 46 Range: 27–62 NR (*n*= 1)	M = 4 F = 7	Lived Experience= 4 ACCHO= 1 Clinician= 4 Other*=2	Urban NT= 6 (Darwin and surrounds) Remote NT= 5 (Alice Springs = 4, Tennant Creek= 1)	441

Abbreviations: ACCHO, Aboriginal Community Controlled Health Organisation; F, Female; M, Male; NR, Not reported; NT, Northern Territory.

*Other encompasses peak body representatives and public health executives

### Themes

Seven global themes were developed: (1) Illicit drug use and harms are diverse and distinct, (2) Client support needs are complex and influenced by co-morbidities, socio-demographic and cultural factors, (3) Priority population sub-groups need targeted strategies, (4) Local service strengths can be further developed and enhanced, (5) Local services need better resourcing, (6) Invest in progressive legislative and policy reforms, and (7) Improve routine monitoring and evaluation. Themes 1–3 relate to patterns and factors that influence illicit drug use and harms, and themes 4–7 relate to service needs and sector priorities. Themes were tabulated (Table 3). Each global theme and underlying organising themes are described below with illustrative quotations.

**Table 3:** Thematic analysis

Global Theme	Organising Themes	Basic Themes
Illicit drug use and harms are diverse and distinct	Patterns of use and harms	High levels of illicit drug use and harms across the Northern Territory despite variable supply and availability across a broad spectrum of illicit drugs
		Binge and problematic illicit drug use patterns observed around major events, local events, and cultural events/ceremony
		Introduction of novel substances linked to population movement into the Northern Territory
	Illicit drugs of concern	Cannabis (‘Gunja’)
		Crystal methamphetamine (‘Ice’)
		Volatile substances and non-beverage alcohol
Client support needs are complex and influenced by co-morbidities, socio-demographic and cultural factors	Supporting a population with complex needs within the constraints of illicit drug services	Navigating social, demographic and cultural determinants of illicit drug use and harms
		Managing mental health conditions co-occurring with illicit drug use and harms
		De-stigmatising illicit drug use and harms
		Addressing poly-drug use
Priority population sub-groups	Equity and access challenges associated elevated by social, demographic and cultural factors	Aboriginal and Torres Strait Islander people and communities
		Young people
		Transient and/or homeless (“long-grassers” and “river people”)
		Those living in rural and remote areas
		Socio-economically disadvantaged
		Those impacted by the criminalisation of illicit drug use
Local service strengths can be further developed and enhanced	Strong investment in residential rehabilitation, and Aboriginal and Torres Strait Islander-led, peer and lived experience-led models of care	Strong investment in residential rehabilitation
		Culturally appropriate care led by the Aboriginal community controlled sector
		Innovative models of care underpinned by peer and lived experience approaches
Local services need better resourcing	Better resourcing, particularly for increased preventative health efforts, harm reduction, and wrap-around support for clients	Better resourcing of the preventative efforts to minimise illicit drug use and harms
		Expansion of residential rehabilitation focusing on illicit drug treatment and aftercare
		Scaling up and further investment in outreach services
		Better resourcing to address social and cultural determinants of illicit drug use and harms
		Further resourcing for targeted support of priority populations
	Greater care innovation, integration, collaboration and service coordination	Explore opportunities for greater specialist input
		Increase resources and the availability of psychological support and trauma-informed counselling
		Greater clarity in definitions of the dual diagnosis of mental health disorders and drug use disorders
		Greater integration between alcohol and other drugs services and mental health services
		Greater collaboration, de-fragmentation and coordination of services
	Increasing capacity for quality and accessible therapeutic responses	Opportunity to increase therapeutic responses to illicit drug use, particularly in the prison system
	Strengthen workforce capacity and capabilities	Attracting and retaining skilled illicit drugs workforce
		Developing workforce skills and capabilities
		Expanding workforce size
		Increasing workforce effectiveness
Invest in progressive legislative and policy reforms	Decriminalising illicit drug use and prioritising a public health/harm reduction approach	Focusing on policy and legislation that prioritises illicit drug harm reduction
		Decriminalising equipment that reduces harms associated with drug use
	Strategic investments and policy development	Development of Northern Territory illicit drug strategy
		Advancement of illicit drug-related evidence base in the Northern Territory
		Development of locally relevant evidence-based practice guidelines
Improve routine monitoring and evaluation	Monitoring and evaluation of illicit drug use, prevalence and impacts	Scale up and enhance routine data collection, monitoring, evaluation and research strategies
		Leverage existing structures and stakeholders to advance processes and knowledge creation

### Illicit drug use and harms are diverse and distinct

Participants expressed that the most concerning illicit substances were cannabis (‘Gunja’), and crystal methamphetamine (‘Ice’) due to their widespread use and patterns of significant harms. One participant noted that cannabis use in the NT is “*very rampant*” and is viewed as a “*social norm*” in the community *(P19*,* Lived Experience*,* Urban NT)*. The social harms associated with cannabis use were frequently mentioned, and harms among Aboriginal and/or Torres Strait Islander population were influenced by cultural practices:

*“Financial harm is a big one*,* a lot of people can’t afford to continue to use it [cannabis]*,* and there’s social impacts of that. In the Aboriginal community*, we see a lot of humbugging* [*cultural practice of sharing resources,* at times*,* in an unreasonable way] for money for cannabis use*,* which then creates a divide within the community. That’s probably the biggest social impact*,* which is derived from a financial impact….” (P8*,* Service Provider*,* Urban NT)*.

Methamphetamine use was associated with harms, often resulting in contact with the criminal justice system. One participant said:

*“…when a complex trauma history intersects with high level methamphetamine use*,* it’s often very explosive*,* and that’s when people end up in the prison system and treatment services.” (P7*,* Service Provider*,* Remote NT)*.

Participants highlighted the burden that methamphetamine use places on the public health system, noting its association with increased hospitalisation risk. In the NT, the population is geographically dispersed. Some communities are many hours away from hospitals by road, and during the Wet season (November-April), many northern NT communities become inaccessible by road. As a result, individuals impacted by Ice use are often transported by air to hospitals, leading to costs to the health system. A different participant said:

*“The anti-social nature of Ice use means it has huge ramifications. Someone that’s intoxicated with Ice is much more likely to end up the emergency department*,* violent criminal activity and with police involvement. That’s having a huge burden on the public health system because we’re seeing higher rates of hospitalisations and need for care flights/transport for drug-related violent crime. Ice and cannabis would be the two central pillars of problems in the community. (P12*,* Other*,* Urban NT)*

Participants described a diversity of illicit drug use and harm patterns, and many noted that some patterns were distinct from what is commonly observed in other Australian regions. Despite the focus on illicit substances in this study, some participants emphasised that reducing alcohol use and harms should be a higher priority than illicit substance use and harms in the NT. Furthermore, inhaling volatile substances (e.g. sniffing aerosols, paint removers) and consumption of non-beverage alcohol (e.g. drinking hand sanitizer, methylated spirits) were identified as significant concerns due to the tendency to be used by young people and those socioeconomically disadvantaged, combined with the risk of significant long-term harms from use.

*“The demographic of people sniffing substances tend to be young people*,* Indigenous almost exclusively*,* from a low socioeconomic demographic with poor social supports. Non-beverage alcohol is a significant issue*,* particularly in Central Australia [remote NT]*,* across an Indigenous demographic*,* and those who are homeless or have limited income.” (P17*,* Clinician*,* Remote NT)*.

Participants reported that illicit drug use trends and harms were influenced by variability in drug supply and availability. Participants who were service providers in the NT’s urban centres reported observing increases in binge and problematic illicit drug use, coinciding with major events, such as music festivals and sport. These events attract people from interstate and are reported to result in the introduction of substances to the community that are less commonly available.

*“When we have music festivals or motorsport events*,* where people are coming from interstate*,* we’re seeing more novel substances used within our community.” (P15*,* Clinician*,* Remote NT)*.

Events like funerals and ceremonies were also associated with upticks in illicit substance use and harms and were associated with population movement within the NT.

*“Visits to town from remote communities often coincide with things like funerals. It’s very much a binge while we’re here thing*,* similar for people living in town. If there’s an event on*,* then there’s a binge*,* it’s just an expectation.” (P16*,* Clinician*,* Remote NT)*.

### Client support needs are complex and influenced by co-morbidities, socio-demographic and cultural factors

Participants described a wide range of support needs for people who are impacted by illicit drug use and harms in the NT. For Aboriginal and/or Torres Strait Islander people, care is best aligned with culturally-informed constructions of health and wellbeing, such as connectedness to spirit, culture, and Country. The determinants of harmful illicit drug use in the NT are often related to these factors and are distinct from those in other Australian states and territories, as one participant said:

“*The issues that underpin harmful [illicit drug] use in the NT are not universal across the country” (P7 Service Provider*,* Remote NT)*.

Participants described how Illicit drug use in the NT produces harms in a more significant way when intersecting with social challenges which are more commonly experienced in the NT, including low income, marginalisation, remoteness, housing instability, and trauma. One participant said:

*“…[in the NT] the vulnerabilities associated with illicit substance use are exacerbated… the people that are already very vulnerable*,* homelessness*,* fixed income*,* trauma*,* mental health issues*,* homelessness*,* significant relationship issues*,* legal issues*,* tend to be the people that will present to places like Emergency Departments and AOD services*,* either in situational relationship crisis*,* or with legal issues that they want to address. Those people tend to experience the harms from substances exponentially compared to people that live in a stable*,* appropriate*,* supportive*,* stable relationship*,* income assured environment.” (P17*,* Clinician*,* Remote NT)*.

Service providers discussed the challenges of supporting people impacted by illicit substance use and harms with complex socio-cultural needs. Deepening poverty rates in the NT, including in remote areas with long-term chronic poverty, exacerbates these challenges. Addressing clients’ complex needs includes delivering strengths-based care and navigating socio-demographic and cultural determinants of use and harms. One participant said:

*“We have clients with housing instability*,* financial issues*,* really significant social determinants that are just not being met in their life.” (P8*,* Service Provider*,* Urban NT)*.

Cultural influences were viewed to normalise cannabis smoking among Aboriginal and/or Torres Strait Islander people, especially in remote communities of the NT. One clinician explained:

*“Culturally*,* smoking ceremonies are a cleansing and a positive thing*,* it’s not seen as a harmful or negative thing… at least 50% of the Indigenous population are smoking cigarettes*,* there’s not a social stigma attached with cannabis use… There’s a sense that in remote communities that cannabis use is normalised*,* seen as not harmful and as a predominantly accepted substance unless somebody experiences a psychosis or behaves in a violent or abnormal way.” (P17*,* Clinician*,* Remote NT)*.

However, service providers described drawing upon cultural protective factors in the planning and delivery of services for Aboriginal and/or Torres Strait Islander people living in remote NT, which led to better outcomes. One service provider in Central Australia described the connection to Country and community, and Aboriginal and/or Torres Strait Islander leadership:

*“In remote communities*,* asking people to go off Country*,* to come to town to attend rehabilitation… there’s whole a lot of issues that come with that… If you got people on their own Country*,* with Aboriginal people from that community running those programs*,* you will find a higher success rate than having them in town.” (P7*,* Service Provider*,* Remote NT)*.

Participants reported co-occurring illicit drug use and mental ill health as a major factor increasing the complexity of client support needs. However, many services were described to lack the resources and/or expertise in the NT system to support people who use illicit drugs with clinically challenging mental health conditions. Participants described a lack of available qualified staff in the NT and having to compete with other health sectors for recruitment, especially for skilled staff who identify as Aboriginal and/or Torres Strait Islander. One participant said:

*“AOD services are not being properly prepared or resourced to manage higher acuity mental health conditions…AOD clients have a very high propensity of low acuity mental health issues that are expected to be managed within the AOD treatment model. The higher acuity… bipolar*,* borderline personalities disorders… usually require a higher level of skill set or therapeutic interventions than what is available in our AOD services. Most of our AOD services don’t have ready access to professionally trained mental health clinicians.…we’ve seen a few situations recently where that has led to clients being returned to emergency departments or pushed back into secure treatment facilities because they weren’t able to be managed in AOD services.” (P1*,* Other*,* Urban NT)*.

As noted above, clients may be diverted from an AOD service due to complex mental health co-morbidities. Other participants illustrated how social perceptions can stigmatise people, leading to reduced access to care in AOD services for clients with co-occurring substance use and mental health conditions:

*“There’s a real risk of AOD patients being ignored*,* stigmatised and marginalised because of the perception of being difficult or complex*,* or having a personality disorder….” (P12*,* Other*,* Urban NT)*.

### Priority population subgroups need targeted strategies

Six priority population subgroups were identified from the analysis by researcher consensus and are listed below in no specific order. One participant summarised the challenges of supporting priority subgroups in the NT as *“it’s an equity and an access issue”* (*P12*,* Other*,* Urban NT)*. Participants felt that targeted strategies and resourcing are needed to effectively support each group.

#### Aboriginal and/or Torres Strait Islander people and communities

Participants highlighted that complex social and cultural factors influence illicit drug use and harms within this population. Such harms are exacerbated by inadequate access to culturally appropriate support. One participant said.

*“Aboriginal and/or Torres Strait Islander populations have high levels of use in the NT… We’d be looking at social determinants and people’s personal reasons for use. There’s cultural reasons why cannabis is more widely accepted socially than other drugs.” (P1*,* Other*,* Urban NT)*.

#### Rural and remote residents

Lack of resources for illicit drug prevention and treatment in these areas resulted in local service gaps. A lived experience advocate described how in these areas, some community members tend to take ownership of early intervention and prevention initiatives, but this is challenging and often of limited effectiveness due to the lack of support and expertise, they said.

*“In remote communities*,* the illicit drug problem is significant. The health clinics and communities are under resourced… unless something really bad happens and somebody has a drug induced psychosis or an attempted suicide… in a community where everyone is smoking gunja*,* it’s up to the community members and the families to do the brief intervention and the preventative work and try to educate their family… It’s very hard to do as there’s no education being put into the communities about illicit drugs.” (P19*,* Lived Experience*,* Urban NT)*.

#### Young people

Participants felt there was inadequate coverage of youth illicit drug services across the NT. Early intervention efforts are critical to prevent illicit substance use disorders, addiction, acute and long-term harms. They described incidences of observed youth drug use in the context of trauma and/or mental ill-health and a lack of meaningful community engagement opportunities, especially in rural and remote areas of the NT. Cannabis use among young people was highlighted as an explicit concern due to social and cultural factors and its higher accessibility compared to other illicit substances. One clinician said.

*“There is a lot of cannabis use from quite early ages*,* a lot of introduction to use*,* and normalisation of use at a young age*,* through generations. Older relatives use so it’s more normalised for younger people to use.” (P14*,* Clinician*,* Remote NT)*.

**Socio-economically disadvantaged people**: Poverty adds complexity to treatment planning, highlighting the need for targeted person-centred support for this group. People in this group often faced multiple challenges such as unstable employment, inadequate housing, and food insecurity, adding complexity to residential treatment aftercare planning. In the interview data, socio-economic disadvantage frequently intersected with remote living, due to the higher drug costs in very remote areas of the NT. One participant described the challenges of experiencing socioeconomic disadvantage in very remote NT, leading to a cycle of harms within families and communities, and exacerbating health inequalities.

*“The price of gunja in [urban NT] 0.5 of a gram is $30 AUD…if you go to [very remote NT] it is $100 AUD…families in remote communities are experiencing living below the poverty line. How can they be able to afford to have a substance use addiction when there’s no money coming in to support their families? You see in the remote clinics*,* kids that have failure to thrive and malnutrition because families don’t have the food to feed their kids because they’re spending their money on addiction.” (P19*,* Lived Experience*,* Urban NT)*.

#### People in contact with the criminal justice system

Participants reflected that having illicit drugs criminalisation policy and legislation often led to greater harms, including the marginalisation and stigmatisation of people who use illicit drugs. This was believed to contribute to a cycle of disconnection from support networks and greater engagement in crime. Additionally, there is a lack of access to culturally appropriate therapeutic care for illicit substance use disorders among individuals within the NT prison system. Some participants called for a more nuanced public health, social justice and decriminalisation approach to illicit drug use.

*“Prohibition and criminalisation of drugs (and illicit alcohol) are a vital consideration as a primary driver of crime*,* stigma*,* treatment hesitancy and adulterated supply. A significant amount of the harms caused by AOD can be attributed to this and we must start treating AOD use as a health concern not a criminal one.” (P1*,* Other*,* Urban NT)*.

#### Transient and/or homeless people

Participants noted an absence of tailored harm minimisation outreach support services for this cohort. Commonly referred to as “long-grassers” and “river people”, they often reside in camps or shelters situated on the outskirts of towns, in areas with long grass (Northern NT), or in dry riverbeds (Central Australia). One participant felt that blood-borne virus transmission was a significant risk in this population due to these living conditions and the limited access to clean needles and syringes. This perception was also attributed to the lack of engagement with public health interventions aimed at promoting safe equipment and the inadequate capacity for services to undertake outreach initiatives. One participant said.

*“There’s a large transient homeless or semi-homeless population in the NT. If we think about the ‘long grass’ population… we haven’t had sophisticated services to deal with that. In a lot of Eastern states*,* there are homeless outreach teams*,* and we clearly don’t do that. We’ve got a transient population*,* which is often hard to engage with and monitor.” (P12*,* Other*,* Urban NT)*.

### Local service strengths can be further developed and enhanced

#### Residential rehabilitation

Participants noted that investment and demand for residential rehabilitation facilities is strong compared to other states and territories.

*“…we have very high rates of residential rehabilitation beds*,* about 5 to 10 times higher than any other Australian jurisdiction. We’ve got a lot of investment in residential beds*,* but we’ve got a relatively small investment in other modes of treatment that are more heavily invested in other states.” (P1*,* Other*,* Urban NT)*.

However, residential rehabilitation services in the NT are generally more explicitly tailored towards alcohol, rather than illicit substances. Participants reported illicit substance use in combination with alcohol (polydrug use) being extremely common in treatment contexts, and this was difficult to *difficult to screen for and assess*, posing challenges for treatment. When asked about the extent to which illicit substances are the primary drug of concern among patients in an NT residential rehabilitation facility, one participant said:

*“I would say almost none. It would be only in the setting of poly drug use. I don’t think we ever have anyone impacted by purely one drug… overall*,* the vast majority of harm is caused by alcohol. That just blows everything else out of the water. As far as I’m concerned*,* every single day*,* we see alcohol-related harm. Illicit drugs clearly contribute to harm*,* at times*,* alcohol and illicit drugs go together*,* and that may not necessarily be apparent to begin with.” (P4*,* Clinician*,* Remote NT)*.

#### Culturally appropriate care led by the Aboriginal community controlled sector

Aboriginal Community Controlled Health Organisations (ACCHOs) are primary health care services that are initiated and operated by the local Aboriginal and Torres Strait Islander community to deliver holistic, comprehensive, and culturally appropriate health care to the community which controls it, through a locally elected Board of Management. Participants noted that ACCHOs are crucial in providing drug harm reduction support to remote community members and often operate independently of local government health systems:

*“There’s a lot of people who inject drugs in remote NT. There’s an afterhours dispensing unit operated by [ACCHO]*,* but they don’t report to government*,* and they don’t particularly want any involvement from them. So*,* they just want to go along and quietly operate it… as long as it’s there*,* that’s a great thing” (P2*,* Other*,* Urban NT).*

Aboriginal community controlled rehabilitation services provide cultural support and an understanding of people’s ways of knowing and being, and deliver treatment as a collective:

*“In the residential rehabilitation service [ACCHO]*,* there’s a real intention to create more cultural engagement and activities based around culture*,* rather than just people sitting there*,* isolated*,* detoxing. The Aboriginal health services are doing it better because they’re actually able to see people in community.” (P5*,* Clinician*,* Remote NT)*.

Services that are led by Aboriginal-identified people were said to use cultural knowledge to design care that represents and respects Aboriginal people’s cultural, community and social structures:

*“There are some real strengths in our Aboriginal staff… For some of the younger clients*,* it’s about teaching them about culture… We have separated men and women’s area. They never come together on site.” (P10*,* ACCHO*,* Remote NT)*.

#### Innovative models of care underpinned by peer and lived experience approaches

Capturing and understanding lived experience voices will inform service models to better support AOD users’ recovery journeys and reduce public and system stigma to increase access. One participant said that *“The strengths of services… they use lived experience.” (P21*,* Lived Experience*,* Urban NT).* Peer and lived experience approaches created connectedness, belonging and empowerment:

*“The outcome of having peers connected to this program is that we’ve seen people with long term challenges beyond their Hep[aitis] C*,* and we’re addressing them. We’re developing rapport with people that have neglected other areas of their health for so long*,* because they’ve finally had a positive experience in a health setting. That’s given them the confidence to talk to someone about some other areas of their health.” (P18*,* Lived Experience*,* Urban NT)*.

A different participant highlighted the importance of developing frameworks that allow services to iterate on their models with consumer-led storytelling as innovative datasets:

*“There are opportunities to learn from people who’ve gone through those experiences*,* what’s helped them overcome their harmful use*,* what’s kept them in recovery if they’re actively in recovery*,* and also*,* what the barriers have been along their journey. My organisation sought to develop understanding through the stories led by people with lived experience trying to gather information about their journeys… to develop what will be a framework for being able to digest people’s stories and draw out the relevant elements…” (P1*,* Other*,* Urban NT)*.

One participant overseeing a service with lived experience and peer models of care emphasised the importance of engaging with lived experience representatives at a safe and secure time in their recovery journeys. However, these requirements may not be reflected across the wider peer workforce:

*“You must be 12 months clean before you can come and work with us. We have employed people before that time frame that has been too early*,* because they’ve had relapses. " (P7*,* Service Provider*,* Remote NT)*.

Being a lived experience representative is a relational role shaped by personal experiences which are used to relate to other community members. These interactions can expose lived experience staff to substance use triggers, so it is important to provide training, mentoring and mental health supports that are guided by evidence-based practice guidelines.

*“Make sure that you’re not setting people’s triggers or traumatising. Make sure that they’ve reached the end of their treatment. Don’t throw people back in at the deep end too quickly because triggers are there. You need to have good theory and practice before you jump back in… Agencies should have clear guidelines this (P10*,* ACCHO*,* Remote NT).*

### Local services need better resourcing

Five key areas of focus were identified by researcher consensus from the analysis to improve illicit drug services in the NT. They were:

#### Better resourcing, particularly for increased preventive health efforts, harm reduction services and wrap-around support for clients

Due to significant complexity in client needs, participants called for greater resourcing for services across the board, including wrap-around services that support substance use determinants.

*“There is a need to better fund and better resource across the spectrum of services… (P14*,* Clinician*,* Remote NT)*.

Without additional funding, the reach, engagement and effectiveness of services that provide harm minimisation support are limited. Participants described how the system currently is skewed towards treatment of people who use illicit drugs in presentations of acute crisis where significant harms have already been experienced, rather than prioritising well-resourced prevention and person-centred care.

*“There is absolutely nothing to support families to understand and assist locally… There’s no capability building of family members to manage the stress that they’re going through*,* to manage their own mental health*,* but also to have an influence on that person or their ability to help that person when they are ready for change.” (P20*,* Lived Experience*,* Urban NT)*.

One participant explicitly attributed insufficient resources to the constraints faced in pursuing harm reduction initiatives:

*“The challenge is trying to provide harm reduction services… It’s expensive and we struggle. We’ve had staff cuts recently… We’re on the bones of our arse financially.” (P2*,* Other*,* Urban NT)*.

While acknowledging its value, a participant called for greater investment to strengthen residential rehabilitation programs across many facets of care. They acknowledged the time, cost, and expertise required to adopt and expand quality improvement initiatives, including monitoring and evaluation functions:

*“There is an urgent need for more funding for residential rehabilitation programs*,* for the venues*,* the quality of facilities and infrastructure*,* for capacity; for quality*,* governance and oversight of program delivery*,* and staffing.” (P14*,* Clinician*,* Remote NT)*.

A lived experience advocate reflected on some of the weaknesses of rehabilitation services in the NT, highlighting the need to focus on aftercare, particularly supporting social determinants that may influence one’s illicit substance use.

*“The weaknesses of the residential rehabilitation services are the program content*,* and just getting in there and getting a bed…. They are also lacking with aftercare. There is no help to get into housing or a job… People need to be supported to go into accommodation and work once they’re at the end of their rehab.” (P21*,* Lived Experience*,* Urban NT)*.

#### Greater care innovation, integration, collaboration and service coordination

One participant described their vital role of Addiction Medicine Specialists in providing outward support across the NT, and there are opportunities to build on this through greater resourcing and coordination:

*“Additional Clinical Addiction Medicine support is critical. They build capacity and can maintain people’s capacity to access [services]*,* harm reduction*,* and good evidence-based support. More Addiction Medicine support could maintain people’s ability to stay safe and get treatment needs met in other settings*,* having a strong core supporting outward is a real need.” (P14*,* Clinician*,* Remote NT)*.

One participant described challenges for coordinating care for patients with high need:

*“It is very*,* very difficult. You’ve got people with complex trauma and nowhere to send them. You’ve got people in crisis and nowhere to send them. You’ve got 6 to 8 year waits in housing and nowhere to send them. It’s really complex. There’s lots of gaps in the Other.” (P8*,* Service Provider*,* Urban NT)*.

A senior public health staff participant recognised the importance of greater definitions for dual diagnosis to concurrently treat mental health conditions and illicit substance use which requires greater integration of AOD and mental health services.

*“I’m keen for dual diagnosis to be clearer*,* with AOD services working with mental health services… Advocacy should bring AOD to the forefront*,* married to mental health*,* while not delineating the two because they’re impossible to treat in isolation.” (P12*,* Other*,* Urban NT)*.

This idea was supported by a different participant, calling for more policy and strategic direction for the management of comorbidity in the NT.

*“There’s a big gap in guiding policy or strategic direction for the management of co-morbidity for the NT. Nationally*,* there’s heaps. Other jurisdictions have really clear policy around how they assign mental health and AOD responsibility*,* treatment*,* and where the clinical divides lie.” (P1*,* Other*,* Urban NT)*.

Many participants emphasised the need for greater collaboration, defragmentation and service coordination. One participant felt that there is an opportunity to support clients who are seeking treatment services to better understand and navigate the system, they said:

*“There are a range of services that are government and non-government*,* they traditionally don’t have strong connections within and across those organisations. There is a need to develop those links… There needs to be a lot of work on articulating*,* defining*,* and advertising*,* the capacity*,* role and pathways for patients to more seamlessly access and integrate across services. If those organisations don’t have good links and understandings*,* it can be very hard and fragmented for someone to navigate through that process.” (P14*,* Clinician*,* Remote NT)*.

Greater collaboration was strained by significant movement in the AOD and public health workforce, and among clients. One service provider described their frustrations:

*“That collaboration to have wrap around services doesn’t really exist. The population here is quite transient*,* making it difficult. If we build a relationship with a specific case manager*,* or even a financial counsellor*,* they’re likely to leave within 6 to 12 months. Then we start again*,* and that can be frustrating. When we do send a referral*,* it feels like it lands on deaf ears” (P8*,* Service Provider*,* Urban NT)*.

#### Increasing capacity for quality and accessible therapeutic responses

Participants discussed significant deficits of access to therapeutic care associated with the prison system:

*“People in the prison system are not getting a lot of good therapeutic interventions… a lot of time is needed to build clients’ understanding of the therapeutic relationship before you can start to get some momentum going around rehabilitation. So*,* if there was more exposure to therapeutic interventions in prison*,* it would be a much better integrated system and process.” (P6*,* Other*,* Urban NT)*.

When therapeutic supports are available, they may not be provided at the right time for the clients’ needs, and this may shift client perceptions of their purpose away from strengths-based principles.

*“People in prison aren’t given the opportunity to go to residential rehabilitation. They get sent to prison and then after a long sentence*,* they go to rehabilitation*,* which is not needed. They need rehabilitation at the start of the sentence. If we want to help people be the best versions of themselves and get clean*,* then we need to be addressing these issues at the start of their custodial sentence instead of at the end.” (P21*,* Lived Experience*,* Urban NT)*.

A participant suggested that increasing the capacity of therapeutic responses could be led by policymakers:

*“Policy and strategy should look at ways… to increase capacity around therapeutic responses that are going to meet people’s needs…” (P11*,* Other*,* Urban NT)*.

#### Strengthen workforce capacity and capabilities

Participants frequently mentioned the significant challenge of attracting and retaining a skilled illicit drugs workforce. Due to this, participants identified the need to strengthen the existing workforce capacity and capabilities by increasing development opportunities and resources particularly focussing on trauma-informed care and mental health related treatment.

*“There’s a huge deficit in trauma counselling and people understanding the causative factors*,* adverse childhood experiences*,* and ongoing adult trauma.” (P17*,* Clinician*,* Urban NT)*.

*“Despite the impact of trauma and trauma type conditions having a strong co-occurrence with substance use disorders*,* there are very few resources available to address those needs.” (P14*,* Clinician*,* Remote NT)*.

One organisation described acting on these gaps, initiating a skill building roundtable:

*“We’re doing a mental health ‘skills build’. We’re having a round table to discuss what units from the Certificate IV [vocational training] in mental health we can put together and upskill people with.” (P3*,* Other*,* Urban NT)*.

Training and skills development can support service providers to advance trauma and culturally informed treatment approaches that represent the localised needs of NT community members, allowing for effective client triaging between services:

*“We need improved skills in the area of personality disorders. We don’t have a lot of highly confident workers in rehabilitation services that know that area well. We could do with some skills in more specific approaches to treatment… trauma-informed*,* culturally-informed*,* best practice*,* cognitive behavioural therapy*,* motivational interviewing. We need good robust baseline skills*,* some additional specialist skills*,* and people can refer on if there’s the need.” (P6*,* Other*,* Urban NT)*.

However, in recognising the need to strengthen workforce capacity and capabilities, one participant identified the high expectations placed on illicit drug service providers. There is an opportunity for providers to be better trained to understand their scope of practice and care coordination opportunities:

*“There are such high expectations on workers in the AOD sector. There’s strong commitment and passion but there’s limitations in what the organisations can do for some clients. Understanding these limitations are important*,* and training around that would be helpful.” (P6*,* Other*,* Urban NT)*.

### Invest in progressive legislative and policy reforms

Participants called for greater focus on legislative changes and decriminalisation of illicit substance use and harm reduction effort, such as the safe use equipment:

*“The more we enter the harm reduction/health promotion/rehabilitation space*,* we’re better for it. When we try to have hardline legislative stances*,* which is*,* ones of complete prohibition*,* high sentencing*,* criminalization of drug use*,* we’re poor for it. The more we accept that*,* and we take a conciliatory approach in our policy*,* the better we are for that.” (P12*,* Other*,* Urban NT)*.

One participant reflected that the current legislation is based on a very limited evidence base and is at times resulting in people engaging in more harmful behaviours:

*“…ball pipes*,* safe smoking kits are totally illegal in the NT. If you get arrested with one and you’ve got previous convictions*,* you’ll go to prison. People know people who have gone to prison for being in possession of a ball pipe*,* so they’re not using it. There’s a cohort of young people… who scare the living hell out of us because they are injecting meth and they’re not injectors. They don’t know what they’re doing.” (P2*,* Other*,* Urban NT)*.

A different participant highlighted the role of policymakers in diminishing structural stigmas and prioritising social justice:

*“There’s so much stigma and shame… Policy and strategy should look at ways to work together in the mental health space to destigmatise…really contextualising this for the NT.” (P11*,* Other*,* Urban NT)*.

Participants called for greater focus on strategic investments into health policy development for illicit drug harm minimisation. One participant called for a comprehensive illicit substance strategy for the NT, and the need for a more developed evidence base to support treatment, including locally relevant co-morbidity guidelines:

*“Ideally*,* we have a comprehensive AOD strategy that reaches across demand*,* supply*,* and harm reduction measures. Recognising that the interplay is there for all of them*,* and they all need to be done properly. As well as*,* the best practice elements for the treatment sector that come from a much more developed evidence base than what we’re currently working on…” (P1*,* Other*,* Urban NT)*.

### Improve routine monitoring and evaluation

Participants called for improved routine monitoring and evaluation to better understand the patterns of illicit drug use and harms in the NT. One participant suggested leveraging existing networks to invest further in quality improvement strategies and the scale up of existing data collection methods:

*“How can systems be improved? The stakeholders are all there in that Harm Reduction Advisory Group… The wastewater testing*,* the illicit drugs reporting system*,* it’s looking at what are the gaps and problems with it. Start with what you’ve got*,* can we scale those up? You’ve already got the people on the ground.” (P11*,* Other*,* Urban NT)*.

At the service level, there is a significant opportunity to improve routine monitoring and evaluation. Service providers face expectations from funders to design and conduct high-quality evaluations, report findings, in turn informing practice, often without sufficient resources to do so. Stakeholders acknowledge the value in working with research organisations to build monitoring and evaluation capacity within their services. One participant said:

*“Funding bodies expect you to be able prove that you are having the impact on the service users that you say you are, and that you’re achieving the outcomes that you intend to achieve… it’s really a complicated space that requires a research brain to be able to work with the service provider*,* and there’s no funding*,* but there’s expectations from the funding body that people justify their funding through outcomes and impacts. The big gaps are A – the funding to do it*,* B – having staff who can perform data collection and manage data inputs and outputs at a level that’s going to give you the data that you need.” (P6*,* Other*,* Urban NT)*.

There is also opportunity to enhance monitoring and evaluation of illicit drug services from a cultural perspective. This includes measuring cultural needs, approaches and outcomes, potentially through a contextually relevant model or tool. One key stakeholder said:

*“…I would like a connection with a research organisation to make sure that we have ongoing evaluation of our service. I rang around*,* ‘who’s got a great model?’ and everyone kind of went*,* ‘we’ve got this model*,* but it still comes back to local issues’… it’s the cultural part*,* how you evaluate that? How do we identify what cultural needs are? Then*,* how do we address that.” (P10*,* ACCHO*,* Remote NT)*.

## Discussion

This study sought to understand illicit drug use, harms, contributing factors and service needs in the NT to inform better service planning and commissioning. The findings address an evidence gap by providing contextual insights into quantitative data, which can help inform legislative and policy reforms, advocacy efforts, and program and service improvements. A key strength of this study’s approach is its ability to capture cultural insights, and this approach can be applied among diverse vulnerable populations worldwide, particularly in areas with limited routine monitoring and evaluation of illicit drug use and harms. Participants described patterns of illicit drug use and harms that were diverse and distinct across the NT, identifying cannabis, crystal methamphetamine, volatile substances, and non-beverage alcohol as primary concerns. These patterns diverge from global contemporary drug trends, which highlight the rise in supply and availability of synthetic substances, including medicinal cannabis, and the non-medical use of psychedelics [7]. Factors influencing illicit drug use and harms included supply, availability, and population movement. In agreement with a recent quantitative report, illicit drug harms were wide ranging [10], and our findings suggested that these harms were influenced by access to treatment and prevention services, client socio-demographic and cultural factors, and health conditions co-occurring with substance use. These results align with international literature investigating illicit drug harms in relation to remoteness, cultural diversity and challenges in accessing health services [34], and emphasise the need for further research on illicit drug use and harms in regional, rural and remote areas worldwide. Participants called for better resourcing to engage priority populations, increased investment in care coordination and integration, enhancement of the overall workforce and therapeutic responses capacity and capability. Identified service needs aligned with the findings from the Demand Study of Alcohol Treatment Services in the NT [14], highlighting the need for further integration of AOD programs and services.

Patterns of illicit substance use in the NT were influenced by socio-cultural factors, and changes in substance availability and affordability, aligning with those observed in displaced populations [35]. Given the high proportion of Aboriginal and/or Torres Strait Islander people living in the NT, this alignment may be attributed to the ongoing impact of colonisation [36]. Nearly 45% of all Aboriginal households live below the poverty line [37, 38]. Such living conditions perpetuate a cycle of employment challenges, education challenges, substance use and harms, and this can only be addressed through investment in social and cultural determinants of health as protective factors. Participants were concerned about observing young Aboriginal and/or Torres Strait Islander people using volatile substances and non-beverage alcohol, in the context of past trauma, socioeconomic disadvantage and a lack of access to other illicit substances. There have been calls to the local government for early intervention responses to minimise and prevent problematic drug use [39]. Study findings support these calls and further investment in the NT to create meaningful opportunities for young people and focus on person-centred harm reduction.

In agreement with emerging international research [40], participants discussed that lived experience roles are highly impactful by providing insights and empathetic support across peer recovery journeys. Lived experience representatives bring an inherently non-judgemental, authentic, recovery-oriented, and destigmatising approach [41]. They also share culture and worldviews with participants, distilling hope, empowerment, and connectedness, which are crucial to mapping a personal recovery journey [42]. In NT recovery services, where 75% of the participants identify as Aboriginal [13], there is a significant need for cultural connection. While participants valued the integration of relational support and personal experience in service delivery, they emphasised the importance of building qualifications amongst the peer workforce, improving workforce readiness, and establishing skilled supervision and supports. These factors have been identified as key strategies to advance NT peer workforce for mental health support [43]. The emerging lived experience service delivery model’s associated risks highlight the need for standardised AOD lived experience guidelines, nationally developed and contextualised for the NT. These should contain specific principles for the illicit drug support workforce to further define and support these roles. The guidelines should be: (1) supported by workforce development in-services, (2) designed to enhance care for people who use illicit drugs, and (3) coordinated by the peak body for AOD support services in the NT, to ensure that both peer workers and their collaborators understand the role, risks and wrap-around supports needed.

A key finding was the need for greater clarity in understanding the role of dual diagnosis, where mental health conditions co-occur with substance use, to address complex needs within diverse treatment contexts. Mental ill-health and substance use comorbidity is frequently reported among people seeking AOD treatment, and is associated with an increased risk of harms, such as suicide attempts, poorer treatment outcomes, contact with the criminal justice system, and premature death [44, 45]. It is estimated that over 47% of the treatment population have a current mental health disorder, and over 33% have multiple mental health conditions co-occurring with their substance use [46–48]. While the data are unclear, the prevalence of dual diagnoses in the NT is likely higher than other Australian jurisdictions because of elevated rates of domestic and family violence, sexual assaults and suicide attempts [49], along with the established link between trauma, substance use and mental ill-health [47]. Participants expressed concerns about stigma and care access barriers for clients with co-morbidity, due to perceived difficulty or complexity. This emphasises the need for further investment in public health destigmatisation and person-centred interventions to raise social and emotional wellbeing and minimising illicit drug use and harms risk before contact with treatment services. Further care coordination and resourcing for specialist input are needed to increase local mental health therapeutic responses. Leveraging the national co-comorbidity guidelines [50] contextualised to the NT, can improve understanding of dual diagnosis among the local AOD workforce.

Going beyond service needs, participants emphasised the need for progressive legislative and policy reforms to reduce harms from illicit drugs. Currently, the NT lacks a specific AOD strategy, with the NT’s 2023-25 Alcohol Action Plan remaining in draft form in June 2024 and omitting illicit drugs, and no separate strategy for drugs in existence. Targeted health and social policies for illicit drugs are needed to complement the NT’s recent alcohol harm minimisation and policy efforts [51, 52], addressing all three pillars of harm minimisation from the National Drug Strategy 2017–2026: demand reduction, supply reduction, and harm reduction [23]. This study supports the full implementation of the National Drug Strategy in the NT by contributing to the evidence base on service needs, illicit drug use and harms. To advance this further, there is a need for improved monitoring and evaluation within AOD services, including through a culturally informed lens, as well broader trends and harms related to illicit drug use. In the absence of a comprehensive NT illicit drugs strategy, advocating for policies and legislation that frame harms from illicit drug use as health and social challenges, rather than criminal issues, is crucial, echoing efforts in the South Australian AOD sector [53]. Moreover, greater investment is needed in developing the NT AOD workforce. The NT AOD Workforce Development Strategic Framework outlines priority action areas to strengthen capacity [54], yet monitoring its implementation has been limited. Future iterations of this Framework should include a focus on the unique needs of the illicit drugs workforce, supported by resources monitoring implementation.

This study had limitations in the sample. Only four participants identified with a lived experience of illicit drug use in the NT, highlighting the need for purposive sampling to amplify the consumer voice, particularly from priority sub-populations, for better health equity. A dedicated study is warranted to better understand these experiences, including those of families and friends, impacted by illicit drug use and harms. Furthermore, there was limited engagement with Aboriginal and/or Torres Strait Islander participants and the community controlled sector, due to tight project timeframes impacting data collection. Future research should have longer lead-in timeframes, underpinned by principles associated with Indigenous leadership, governance and data sovereignty, similar to those undertaken in other jurisdictions [55]. The present study included 10 participants in treatment contexts, potentially skewing the focus towards illicit drug use and harm treatment, rather than prevention, harm reduction and early intervention efforts. However, participants stressed the importance of investing in health promotion and prevention, often framed in relation to illicit drugs harm minimisation strategy development. Despite limitations, study strengths include a geographically dispersed research team across the NT, with strong networks across the AOD sector, aiding project completion within tight timeframes. These factors helped mitigate potential sampling bias by including diverse participants from rural and remote areas, public health, and advocacy roles.

## Conclusions

The patterns of illicit drug use in the NT, as reported by key stakeholders, appears to diverge from global contemporary drug trends. The needs of people impacted by illicit substances in the NT are highly complex, and participants identified local needs and priorities to minimise harms associated with substance use. These include scaling up and building upon the existing strengths in the NT AOD sector, including residential rehabilitation, and culturally appropriate and lived experience models of care. Significant investment is also needed across a wide range of areas, such as integration, collaboration, workforce capacity and capabilities, and strengthening monitoring and evaluation efforts. Findings should inform progressive NT legislative reforms; guide policy reorientation at NT-wide and national levels; and strengthen the commissioning of illicit drugs programs and services at the local level.

## Electronic supplementary material

Below is the link to the electronic supplementary material.

Supplementary Material 1

## Data Availability

The authors confirm that the data supporting the findings of this study are available within the article and its supplementary materials.

## Abbreviations

AOD: Alcohol and Other Drugs
ACCHO: Aboriginal Community Controlled Health Organisation
NT: Northern Territory
NDSHS: National Drug Strategy Household Survey
IDRS: Illicit Drug Reporting System
EDRS: Ecstasy and Related Drugs Reporting System
NTLEN: Northern Territory Lived Experience Network
COREQ: Consolidated Criteria for Reporting Qualitative Research
